# Computer-generated moiré profilometry based on fringe-superposition

**DOI:** 10.1038/s41598-020-74167-w

**Published:** 2020-10-14

**Authors:** Chengmeng Li, Yiping Cao, Lu Wang, Yingying Wan, Hongmei Li, Cai Xu, Hechen Zhang

**Affiliations:** 1 Department of Opto-Electronics, Sichuan University, Chengdu, 610064 China; 2 College of Physics and Engineering, Chengdu Normal University, Chengdu, 611130 Sichuan China

**Keywords:** Optical techniques, Optical physics

## Abstract

A computer-generated moiré profilometry based on algebraic addition instead of algebraic multiplication is proposed. Firstly, the two AC components of the captured fringe patterns on the reference plane with $\pi /2$ phase difference are retrieved and saved in advance. While measuring, two sinusoidal gratings with $\pi$ phase difference are projected onto the measured object alternatively, and the corresponding deformed patterns are captured. Then the AC component of the captured deformed pattern can be separated exactly. When the positive and negative AC component of the captured deformed pattern are added to the two prestored AC components respectively, two moiré fringes only reflect sine and cosine of the object’s phase information can be successfully generated via a series of data processing procedures. Finally, the phase distribution of the measured object can be extracted by arctangent of the ratio of these two moiré fringes. Compared with computer-generated moiré profilometry based on algebraic multiplication, this proposed method can reduce the effect of high frequency noise and residual DC component on measurement and improve the measurement accuracy. While compared with $\pi$ phase shifting FTP, this method can measure more complex objects with better measurement capability. Experimental results verify the feasibility and validity of the proposed method.

## Introduction

Structured light projection three-dimensional (3D) measurement^1–5^ has become one of the important means of 3D measurement because of its unique non-contact, high measuring accuracy, simple operation and other advantages. Among them, phase measurement profilometry (PMP)^6–9^ and Fourier transform profilometry (FTP)^10–12^ are most widely used, and they two have their respective applicable fields. PMP is of higher measuring accuracy. However, it needs at least three frames deformed patterns. This property limits the application of PMP in dynamic and real-time measurement to some extent. Some methods have been proposed to solve this problem. Guan et al. reported a composite phase measurement profilometry^13,14^, in which only one composite fringe pattern is used to recover the object surface. At least three carrier frequencies are used in this method, which inevitably increases the problem associated with spectrum aliasing and reduces the measurement precision. Another single-shot PMP is based on color-encoded grating projection^15,16^. Three gratings with an equivalent shifting phase of $2\pi /3$ are coded into red (R), green (G) and blue (B) channels to synthesize a color grating. When measuring, only one frame grating needs to be projected and captured. Three deformed patterns can be separated from the captured color fringe pattern to retrieve the 3D surface of the measured object. However, due to the problem of color crosstalk among the three channels of R, G and B, the measuring accuracy will be affected. For this, projecting the color-encoded grating rapidly and sequentially by the color wheel of the projector and using a high-speed monochrome camera synchronized with the projector signal to capture the corresponding deformed patterns is proposed^17–19^. This method avoids the problem of color crosstalk, but the gray scale is out of balance, which needs to be corrected in the post processing. FTP can retrieve the 3D shape of the object by using single frame deformed pattern, which owns great real-time and dynamic measurement ability. But the edge and detail information of the measured object always be smoothed to a certain extent due to the filtering operation. For the above situation, a sinusoidal grating projection combined with a $\pi$ phase shifting technique^20^ is come up to eliminate zero-frequency component to improve the measurement precision and range. Chen et al.^21^ presented an improved Fourier transform profilometry based on bicolor fringe pattern, in which two $\pi$ phase shifting gratings are combined into one color fringe. Yue et al.^22,23^ proposed a composite structured light pattern projection method in which the projected grating is formed by modulating two sinusoidal gratings to two distinct carrier frequencies in the orthogonal direction. The former FTP method has the problem of color crosstalk while the later needs to separate two deformed patterns by filtering operation. The measuring accuracy might be not satisfactory enough.

Recently, our group proposed a computer-generated moiré profilometry (CGMP)^24^, which can reconstruct the 3D surface of the measured objects by only one deformed pattern. The experimental results verified that this method has better universality and higher measuring accuracy than FTP. Then, a high-precision computer-generated moiré profilometry (HCGMP)^25^ was proposed. By projecting two complementary gratings and capturing the corresponding deformed patterns, the background light component can be eliminated more accurately. Its measuring accuracy is comparable to that of the four-step PMP. And it was also implemented to apply to the real-time measuring by projector’s time-sharing projection and CCD camera’s synchronous acquisition. On this basis, it is found that the moiré fringes generated by algebraic addition instead of algebraic multiplication can achieve better measurement effect. Because using algebraic addition to generate moiré fringes can effectively reduce the influence of the residual DC component remaining in the AC component of fringe patterns and deformed patterns on the measurement results. The experimental results indicate that the computer-generated moiré profilometry based on algebraic addition superposition has higher precision than both HCGMP and $\pi$ phase shifting FTP.

## Methods

Before measuring, when four sinusoidal gratings with a step of $\pi /2$ phase difference are projected onto the reference plane, four fringe patterns $I_{r1} (x,y)$, $I_{r2} (x,y)$, $I_{r3} (x,y)$ and $I_{r4} (x,y)$ can be captured as shown in Eqs. (1)–(4):

1 $$ I_{r1} (x,y) = R_{0} (x,y)\{ a + b\cos [2\pi fx + \phi_{0} (x,y)]\}, $$

2 $$ I_{r2} (x,y) = R_{0} (x,y)\{ a + b\cos [2\pi fx + \phi_{0} (x,y) + \pi /2]\}, $$

3 $$ I_{r3} (x,y) = R_{0} (x,y)\{ a + b\cos [2\pi fx + \phi_{0} (x,y) + \pi ]\}, $$

4 $$ I_{r4} (x,y) = R_{0} (x,y)\{ a + b\cos [2\pi fx + \phi_{0} (x,y) + 3\pi /2]\}, $$

where $R_{0} (x,y)$ denotes the reflection coefficient of the reference plane, $f$ is the frequency of the fringe pattern on the reference plane, $a$ and $b$ are two constants determined when the projected gratings are generated, and $\phi_{0} (x,y)$ reflects reference plane’s phase information. By subtracting Eq. (3) from Eq. (1), Eq. (4) from Eq. (2), the background light component of the fringes can be eliminated, just as shown in Eqs. (5) and (6):

5 $$ AC_{r1} (x,y) = R_{0} (x,y)b\cos [2\pi fx + \phi_{0} (x,y)] = [I_{r1} (x,y) - I_{r3} (x,y)]/2 ,$$

6 $$ AC_{r2} (x,y) = R_{0} (x,y)b\cos [2\pi fx + \phi_{0} (x,y) + \pi /2] = [I_{r2} (x,y) - I_{r4} (x,y)]/2 .$$

These two AC components are prestored in the computer.

When measuring, two frames gratings with $\pi$ phase difference are projected onto the measured object and the corresponding deformed patterns $I_{o1} (x,y)$ and $I_{o2} (x,y)$ can be captured as shown in Eqs. (7) and (8):

7 $$ I_{o1} (x,y) = R_{1} (x,y)\{ a + b\cos [2\pi fx + \phi (x,y)]\} $$

8 $$ I_{o2} (x,y) = R_{1} (x,y)\{ a + b\cos [2\pi fx + \phi (x,y) + \pi ]\} ,$$

9 $$ AC_{o1} (x,y) = R_{1} (x,y)b\cos [2\pi fx + \phi (x,y)] = [I_{o1} (x,y) - I_{o2} (x,y)]/2 ,$$

where $R_{1} (x,y)$ denotes the reflection coefficient of the measured object and $\phi (x,y)$ denotes the phase information modulated by both the measured object and the reference plane. In the same way, the AC component of the deformed pattern can be obtained as shown in Eq. (9). By adding the positive and the negative $AC_{o1} (x,y)$ with the first prestored $AC_{r1} (x,y)$, two superposition fringes $I_{n1} (x,y)$ and $I_{n2} (x,y)$ as shown in Eqs. (10) and (11) can be generated

10 $$ \begin{aligned} I_{n1} (x,y) &=  AC_{o1} (x,y) + AC_{r1} (x,y) \\ &=  R_{1} (x,y)b\cos [2\pi fx + \phi (x,y)] + R_{0} (x,y)b\cos [2\pi fx + \phi_{0} (x,y)] \\ &=  \frac{1}{2}e^{i2\pi fx} [R_{1} (x,y)be^{i\varphi (x,y)} + R_{0} (x,y)be^{{i\varphi_{0} (x,y)}} ] \\ &\quad + \frac{1}{2}e^{ - i2\pi fx} [R_{1} (x,y)be^{ - i\varphi (x,y)} + R_{0} (x,y)be^{{ - i\varphi_{0} (x,y)}} ] \\ \end{aligned} ,$$

11 $$ \begin{aligned} I_{n2} (x,y) &= - AC_{o1} (x,y) + AC_{r1} (x,y) \\ &= - R_{1} (x,y)b\cos [2\pi fx + \phi (x,y)] + R_{0} (x,y)b\cos [2\pi fx + \phi_{0} (x,y)] \\ &= \frac{1}{2}e^{i2\pi fx} [R_{0} (x,y)be^{{i\varphi_{0} (x,y)}} - R_{1} (x,y)be^{i\varphi (x,y)} ] \\ & \quad+ \frac{1}{2}e^{ - i2\pi fx} [R_{0} (x,y)be^{{ - i\varphi_{0} (x,y)}} - R_{1} (x,y)be^{ - i\varphi (x,y)} ] \\ \end{aligned}. $$

When the positive first order of the spectrum of Eq. (10) is filtered out and multiply with its complex conjugate in the spatial domain, the moiré component with a DC constant (Eq. (12)) can be obtained. Do the same for Eq. (11), the result can be expressed as Eq. (13):

12 $$ \begin{aligned} I_{e1} (x,y)& = \frac{1}{4}[R_{0}^{2} (x,y)b^{2} + R_{1}^{2} (x,y)b] \\ &\quad + \frac{1}{2}R_{0} (x,y)R_{1} (x,y)b^{2} \cos [\varphi (x,y) - \varphi_{0} (x,y)] \\ \end{aligned} ,$$

13 $$ \begin{aligned} I_{e2} (x,y) &=  \frac{1}{4}[R_{0}^{2} (x,y)b^{2} + R_{1}^{2} (x,y)b] \\ &\quad - \frac{1}{2}R_{0} (x,y)R_{1} (x,y)b^{2} \cos [\varphi (x,y) - \varphi_{0} (x,y)] \\ \end{aligned}.$$

The moiré component just containing cosine of the object’s phase information can be obtained by subtracting Eq. (13) from Eq. (12) as shown in Eq. (14):

14 $$ I_{\cos } (x,y) = I_{e1} (x,y) - I_{e2} (x,y) = R_{0} (x,y)R_{1} (x,y)b^{2} \cos [\varphi (x,y) - \varphi_{0} (x,y)] .$$

Similarly, if $AC_{r1} (x,y)$ is substituted by $AC_{r2} (x,y)$, another moiré fringes which just reflecting sine of the object’s phase information can be obtained. The mathematical expressions are shown in Eqs. (15)–(19).

15 $$ I_{n3} (x,y) = AC_{o1} (x,y) + AC_{r2} (x,y), $$

16 $$ I_{n4} (x,y) = - AC_{o1} (x,y) + AC_{r2} (x,y) ,$$

17 $$ \begin{aligned} I_{e3} (x,y) &=  \frac{1}{4}[R_{0}^{2} (x,y)b^{2} + R_{1}^{2} (x,y)b] \\ &\quad + \frac{1}{2}R_{0} (x,y)R_{1} (x,y)b^{2} \cos [\varphi (x,y) - \varphi_{0} (x,y) - \pi /2] \\ \end{aligned}, $$

18 $$ \begin{aligned} I_{e4} (x,y) = & \frac{1}{4}[R_{0}^{2} (x,y)b^{2} + R_{1}^{2} (x,y)b] \\ &\quad - \frac{1}{2}R_{0} (x,y)R_{1} (x,y)b^{2} \cos [\varphi (x,y) - \varphi_{0} (x,y) - \pi /2] \\ \end{aligned}, $$

19 $$ I_{\sin } (x,y) = I_{e3} (x,y) - I_{e4} (x,y) = R_{0} (x,y)R_{1} (x,y)b^{2} \sin [\varphi (x,y) - \varphi_{0} (x,y)]. $$

So, the tangent of the object’s phase information can be obtained just let Eq. (19) divide by Eq. (14):

20 $$ \tan [\varphi (x,y) - \varphi_{0} (x,y)] = \frac{{I_{\sin } (x,y)}}{{I_{\cos } (x,y)}}. $$

The object’s phase distribution $\Delta \varphi (x,y) = \varphi (x,y) - \varphi_{0} (x,y)$ calculated by arctangent is wrapped within $( - \pi ,\;\pi ]$, so the phase unwrapping method^26^ needs to be used here to transform wrapped phase $\Delta \varphi (x,y)$ into unwrapped phase $\Delta \phi (x,y)$. Later, the 3D surface can be reconstructed by phase-to-height mapping relationship^27^ as Eq. (21):

21 $$ \frac{1}{h(x,y)} = a(x,y) + b_{1} (x,y)\frac{1}{\Delta \phi (x,y)} + b_{2} (x,y)\frac{{\phi_{r} (x,y)}}{\Delta \phi (x,y)} + c(x,y)\frac{1}{{\Delta \phi^{2} (x,y)}}, $$

where $\phi_{r} (x,y)$ means the unwrapped phase of the reference plane, $a(x,y)$, $b_{1} (x,y)$, $b_{2} (x,y)$ and $c(x,y)$ are constant coefficients which can be calibrated by some planes with different known heights in advance.

**Figure 1:**
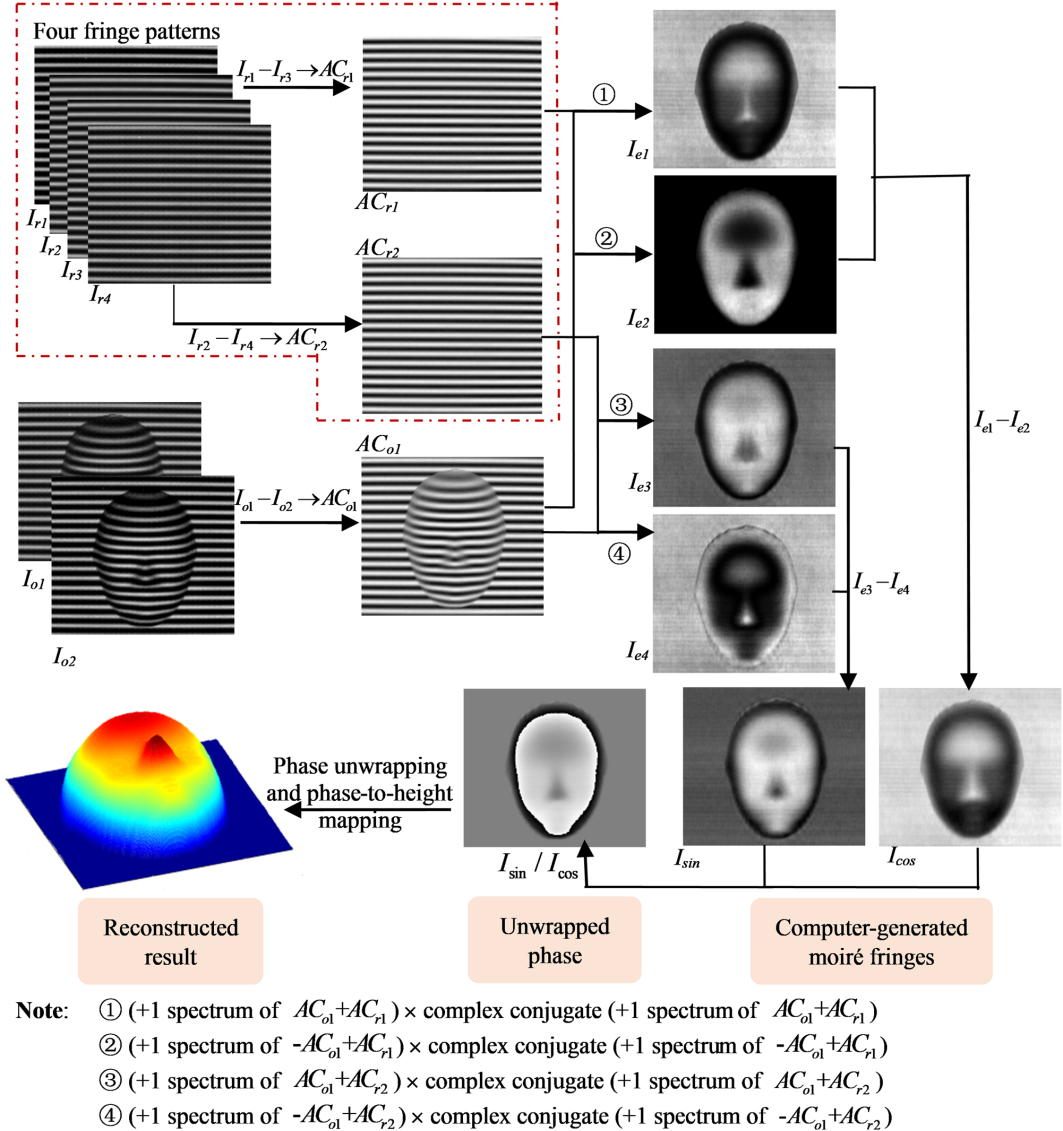
The process flow chart of computer-generated moiré profilometry based on algebraic addition.

The process flow chart of the proposed method is shown in Fig. 1, where the dot line part shows the pre-preparation process.

## Results and discussions

**Figure 2:**
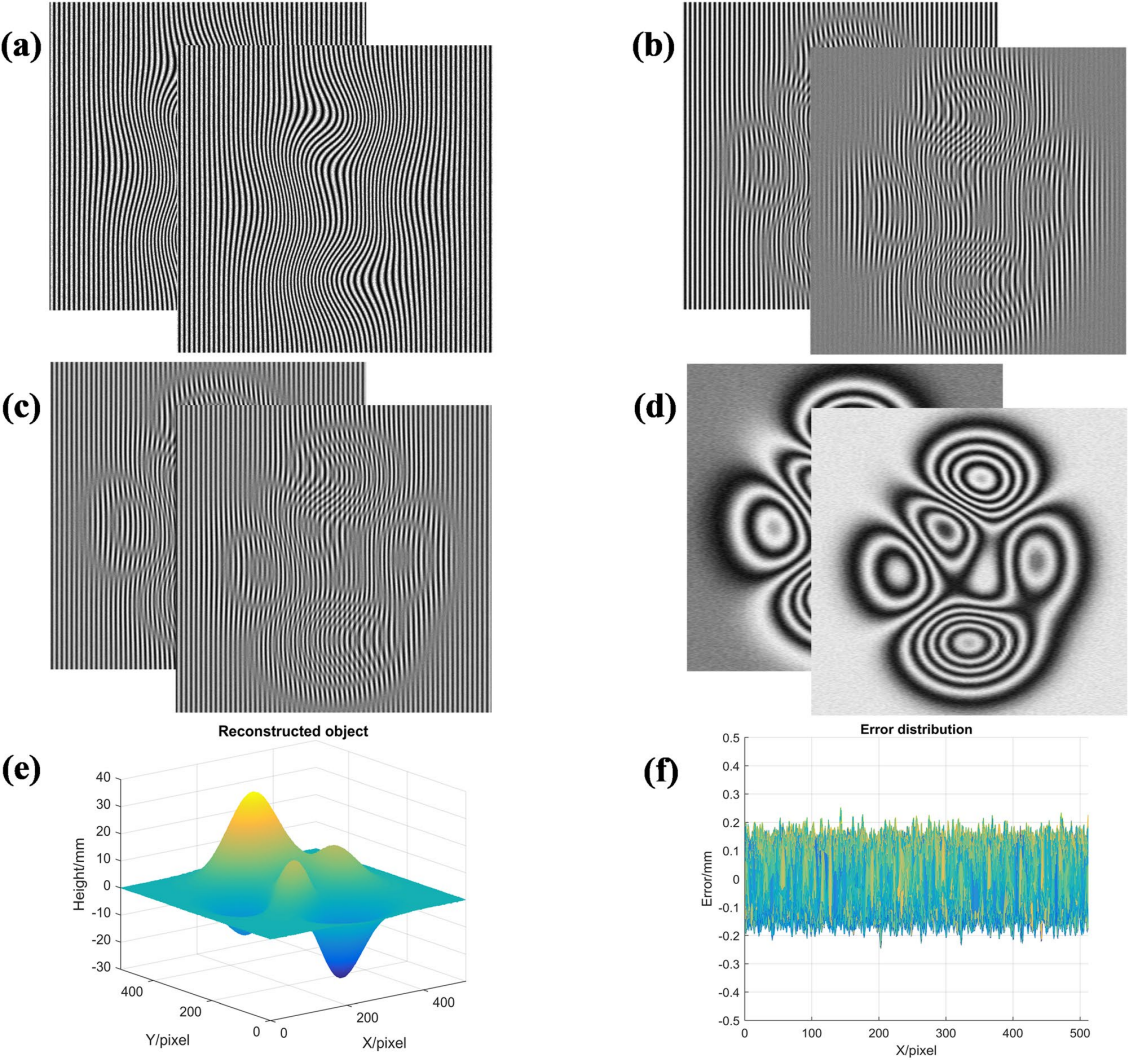
Simulation results of the proposed method: (**a**) two simulated deformed patterns; (**b**) the result obtained by the first deformed pattern and reference fringes; (**c**) the result obtained by the second deformed pattern and reference fringes; (**d**) two generated moiré fringes; (**e**) reconstructed object; (**f**) the error distribution.

To prove the feasibility of the proposed method, a large number of simulation experiments have been carried out. The measurement results of taking the peaks function as the simulated object are shown in Fig. 2. Two simulated deformed patterns with reflectivity distribution reflecting variations in height are generated as shown in Fig. 2a, in which 2% random noise is added to simulate the actual situation. After eliminating the background light component of the first deformed pattern, the superposition fringes as shown in Fig. 2b,c can be obtained by adding the positive and the negative AC component of this deformed pattern with the first and second prestored AC components of the fringe patterns respectively. Two computer-generated moiré fringes obtained from these superposition fringes are shown in Fig. 2d, which represent the cosine and sine of the phase information of the measured object respectively. Phase distribution of the measured object can be obtained by arctangent operation. Finally, the 3D surface of the measured object can be reconstructed by phase unwrapping and phase-to-height mapping. The reconstructed object is shown in Fig. 2e. It can be seen that the proposed method can reconstruct the measured object successfully. The measurement error distribution of the method is shown in Fig. 2f and the error is between − 0.2 and + 0.2. Its absolute mean error (MAE) is 0.0391 mm.

Comparing the simulation results of the proposed method with those of FTP and HCGMP, the comparison result can be obtained as shown in Table 1. The peaks function with noise added is taken as the simulated object, all three methods can reconstruct the 3D surface of the object successfully. Among them, the FTP can retrieve the plane region well, but in the area where the object surface shape changes obviously, there occurs large error. The absolute average error (MAE) and root mean square (RMS) of the result obtained by FTP is 0.0414 mm and 0.0745 mm. The measurement error distribution of the proposed method is similar to that of HCGMP. Their MAE is 0.0391 mm and 0.0399 mm and RMS is 0.0491 and 0.0500, respectively. From the comparison of the simulation experiments, it can be seen that the proposed method has a certain degree of accuracy improvement compared with both FTP and HCGMP.

**Table 1:** Comparison of simulation results of three methods.

Method	$\pi$ phase-shifting FTP	HCGMP	Proposed
Error distribution			
MAE	0.0414 mm	0.0399 mm	0.0391 mm
RMS	0.0745 mm	0.0500 mm	0.0491 mm

**Figure 3:**
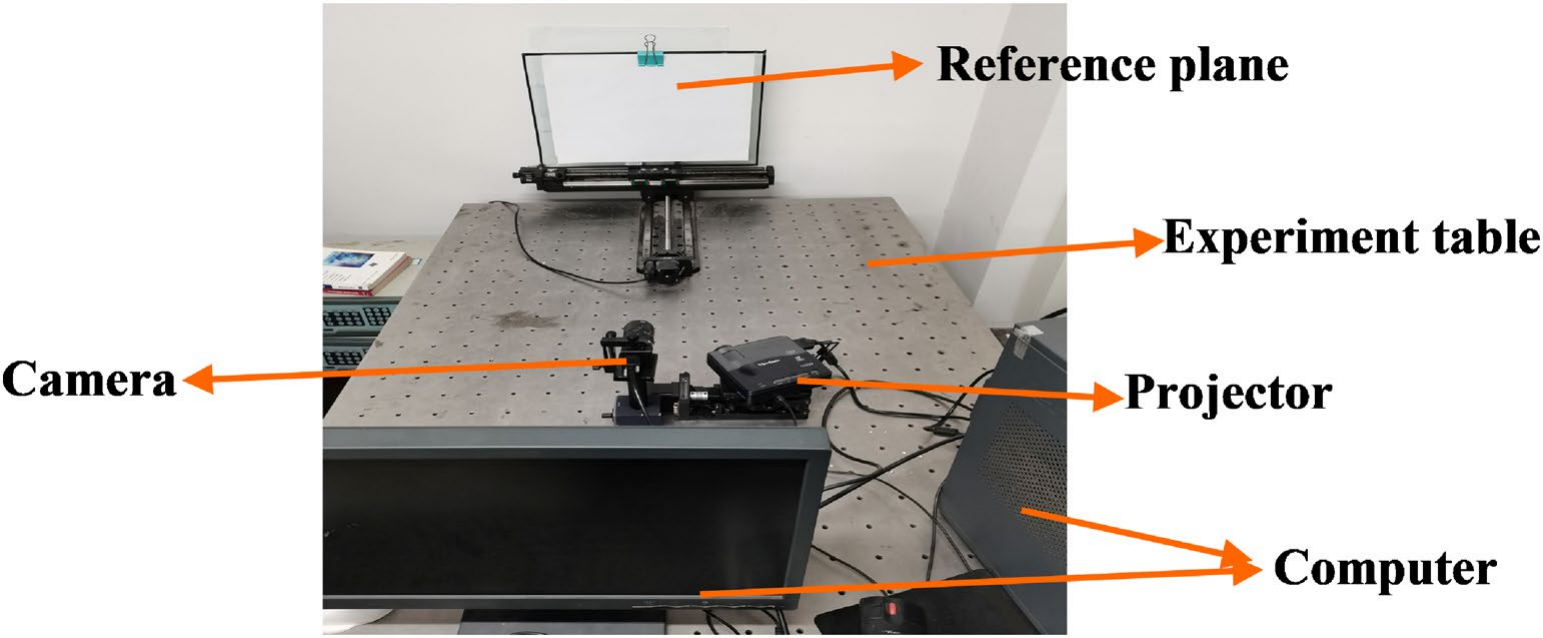
The setup of experimental system.

In order to verify the feasibility of the proposed method in the actual measurement, a series of practical measurement experiments are carried out and the measuring setup is built as shown in Fig. 3. The setup is mainly composed of a CCD camera, a DLP projector and a computer.

**Figure 4:**
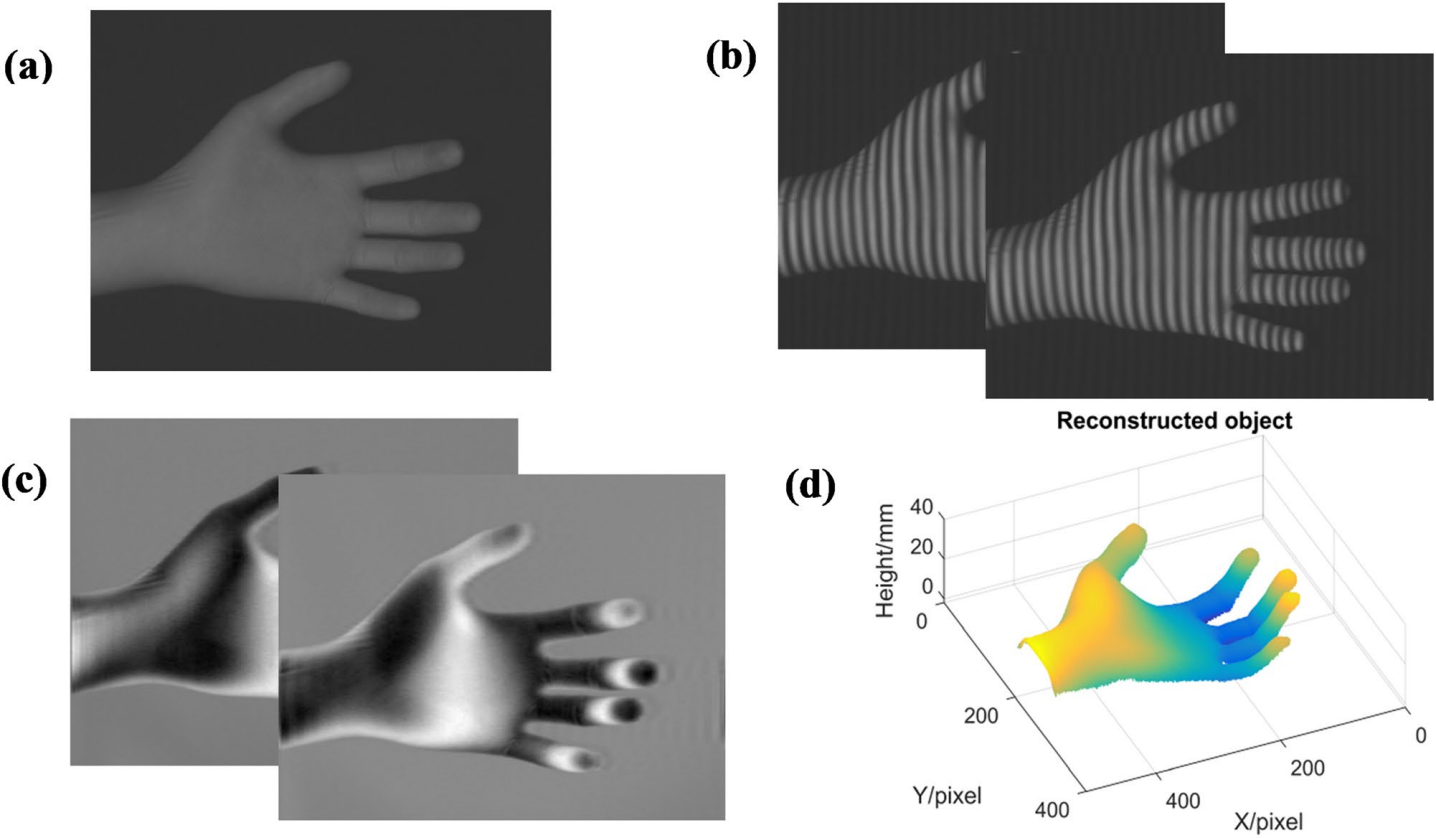
Experimental results of the proposed method: (**a**) the measured object; (**b**) two deformed patterns; (**c**) two generated moiré fringes; (**d**) the reconstructed object.

A palm as Fig. 4a shows is taken to be the measured object. The experimental results are shown in Fig. 4. Two deformed patterns as shown in Fig. 4b are captured to accurately eliminate the background light component. Two moiré fringes can be generated by superimposing the processed deformed pattern modulated by the measured object with the two processed fringe patterns on the reference plane. As shown in Fig. 4c, these two moiré fringes all contain object’s phase information and have $\pi /2$ phase difference from each other. By these two moiré fringes, the phase distribution of the measured object can be retrieved. After using phase unwrapping and phase-to-height mapping, the reconstructed object can be obtained finally as shown in Fig. 4d. It can be seen that the 3D surface of the measured object can be reconstructed well.

**Figure 5:**
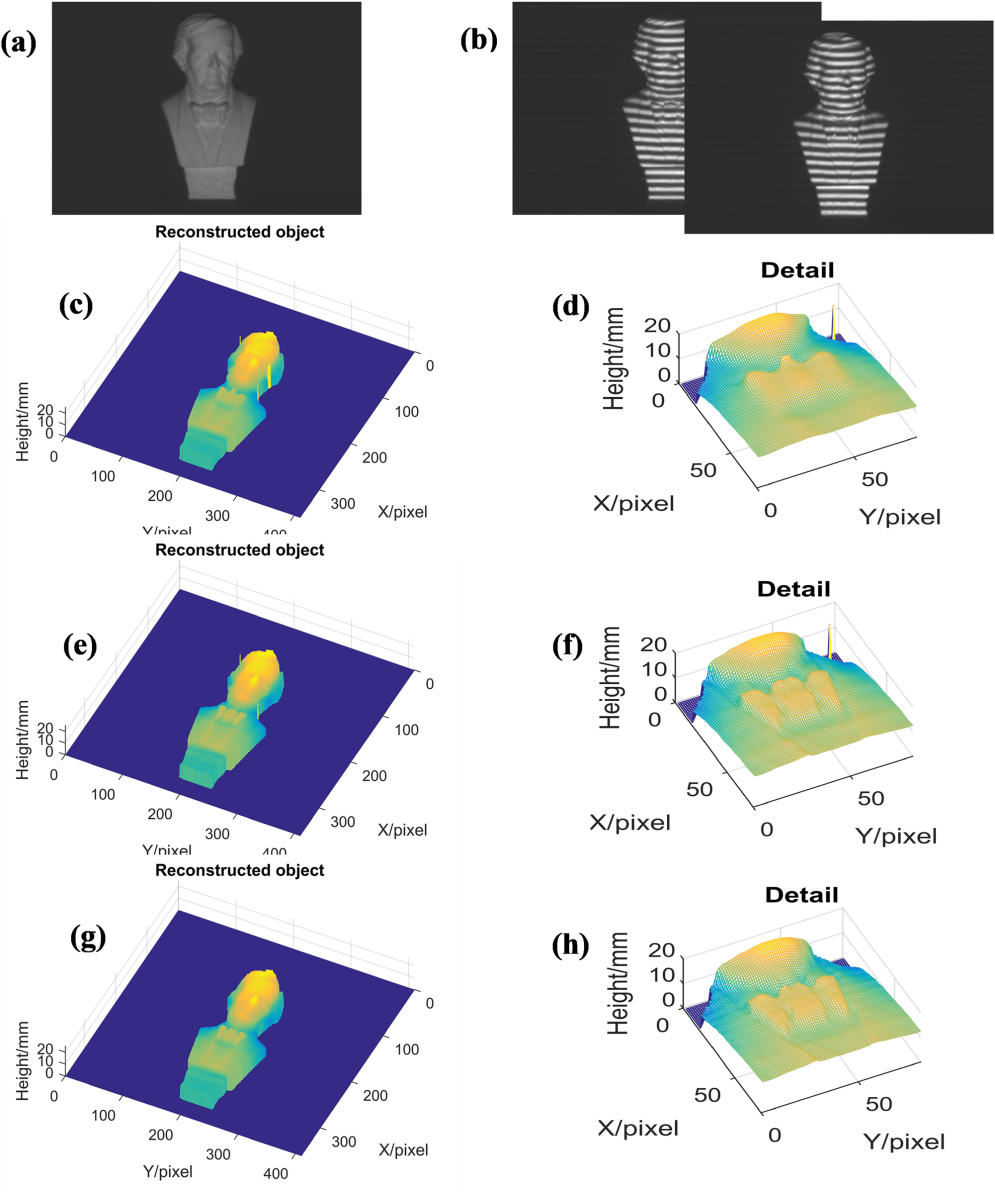
Reconstructed results of complex object by $\pi$ phase shifting FTP, HCGMP and the proposed method: (**a**) the measured object; (**b**) two deformed patterns; (**c**) result reconstructed by FTP; (**d**) enlarged details of (c); (**e**) result reconstructed by HCGMP; (**f**) enlarged details of (e) ; (**g**) result reconstructed by the proposed method; (**h**) enlarged details of (g).

When measuring complex objects, the measurement effect of the proposed method is better than both FTP and HCGMP. To verify this conclusion, a gypsum portrait with rich details is measured. Results of the proposed method are compared with those of HCGMP and FTP. $\pi$ phase-shifting FTP is used to instead of single-shot FTP to guarantee the fairness of comparison. All three methods are based on two frame deformed patterns for phase retrieving. Experimental results are shown in Fig. 5. Figure 5a is the measured object. Figure 5b shows two deformed patterns with $\pi$ phase difference from each other. The reconstructed result of FTP, HCGMP and the proposed method are shown in Fig. 5c,e,g, respectively. It can be seen that FTP and HCGMP have some errors in the measuring result while the proposed method reconstructs the object completely. To show detailed information, the bowknot areas on the chest of these three reconstructed results are enlarged as shown in Fig. 5d,f,h. Obviously, the results reconstructed by $\pi$ phase-shifting FTP show obvious edge smoothing and detail loss, the result by HCGMP also has a little smoothing, while the result by the proposed method are the most complete in detail.

**Figure 6:**
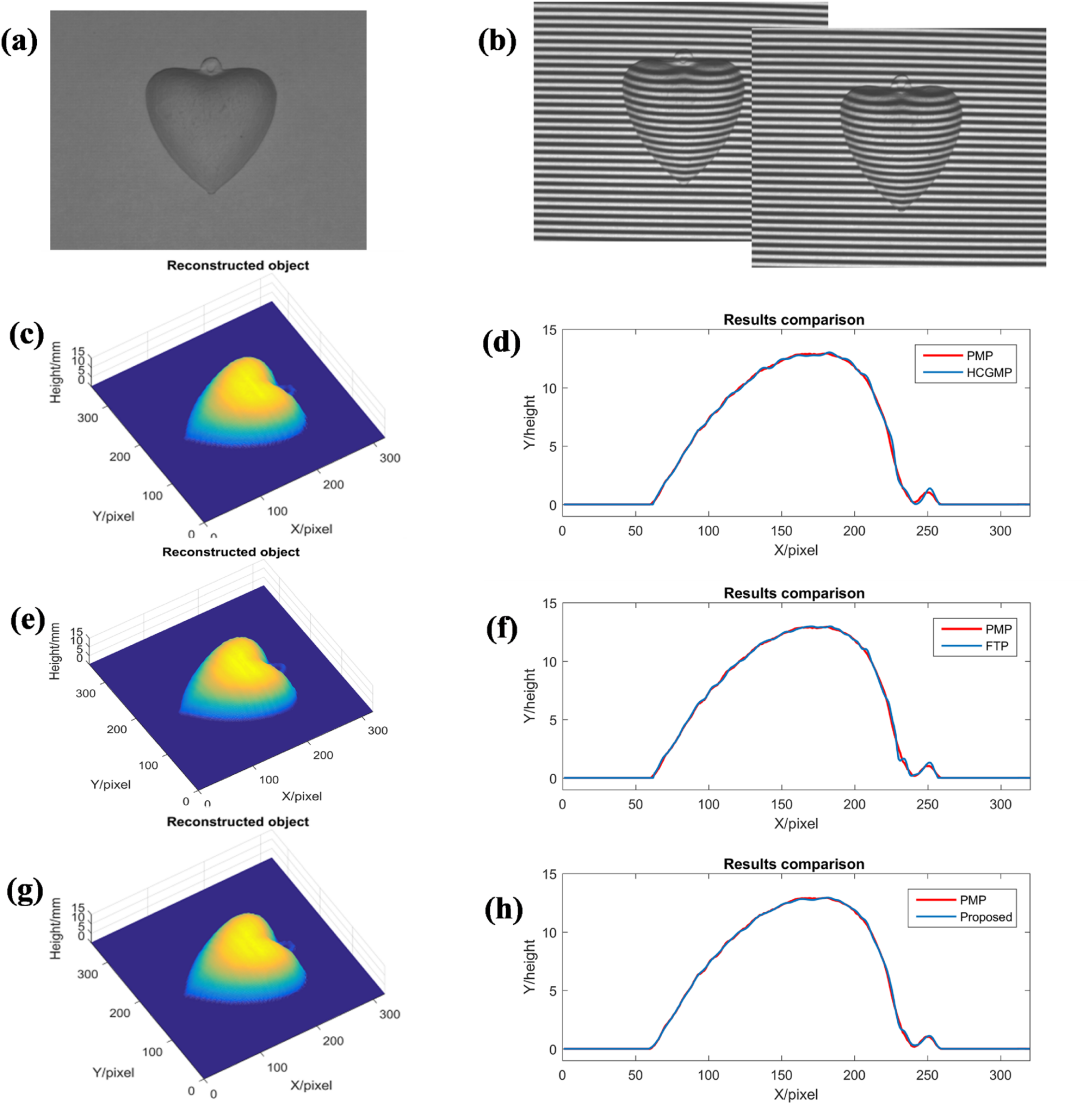
Reconstructed results obtained by HCGMP, FTP and the proposed method: (**a**) the measured object; (**b**) the two deformed patterns; (**c**) result reconstructed by HCGMP; (**d**) results comparison between HCGMP and PMP; (**e**) result reconstructed by FTP; (**f**) results comparison between FTP and PMP; (**g**) result reconstructed by the proposed method; (**h**) results comparison between the proposed method and PMP.

The measuring accuracy of the proposed method is also improved to a certain extent compared with HCGMP and FTP in measuring simple surface objects. The experimental results are shown in Fig. 6. The measured object is a heart model as shown in Fig. 6a. Figure 6b shows the two deformed patterns. Reconstructed results by HCGMP, FTP and the proposed method are shown in Fig. 6c,e,g, respectively. All three methods obtain the 3D surface of the measured object successfully. The result obtained by PMP is taken as the standard because of its high accuracy. For comparison, column 180 of the results obtained by these three methods are extracted and draw in one picture with the same column of the result by PMP respectively, the results are shown in Fig. 6d,f,h. It can be seen that compared with both HCGMP and FTP, the measurement result of the proposed method is closer to that of PMP. Specifically, the filtering operation in FTP always smooths the edge and detail information of the measured object, so that the accuracy is limited. When the moiré fringes are generated by algebraic multiplication, due to the trigonometric properties, some high frequency noise will be processed into zero-frequency component and become interference terms in moiré fringes, thus, the measuring accuracy will be affected. The proposed method of generating moiré by algebraic addition will not introduce this noise into zero-frequency component. Besides, in HCGMP, the residual DC component of both the fringe pattern and deformed pattern will also affect the measurement accuracy. While in the proposed method, the moiré fringes are generated by filtering the positive first order of the spectrum of superposition fringes, this process further reduces the residual DC component and its influence on the measurement results. So, the accuracy of the proposed method can be improved to some extent when compared with the HCGMP and FTP.

**Figure 7:**
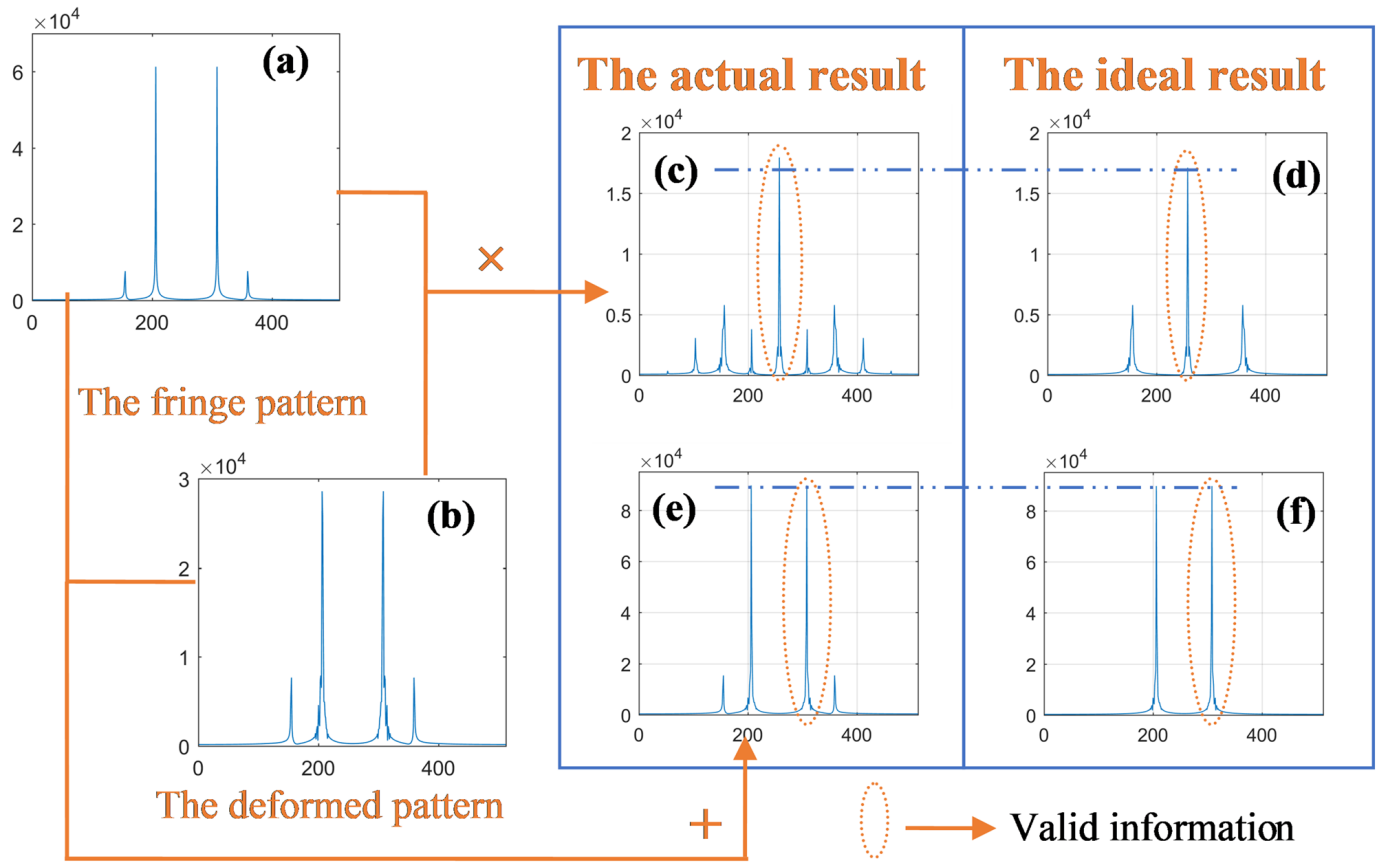
Spectrum comparison of superposed results based on different superposition methods.

Figure 7 further illustrates the difference between the results generated by algebraic addition and algebraic multiplication. When there is high-frequency noise in the reference pattern and deformed pattern, their spectrum after eliminating the zero frequency can be shown as Fig. 7a,b. The frequency spectrum distribution of the result of superposition of these two patterns by algebraic multiplication is shown in Fig. 7c, and the result of superposition in the ideal condition without high frequency noise is shown in Fig. 7d. Comparing the above two results, it can be seen that the zero frequency components (valid information) of the actual result is different from that of the ideal result, which is caused by high frequency noise mixing into zero frequency. In the same case, the result of superposition by algebraic addition is shown in Fig. 7e, and in the ideal condition, the result of superposition is shown in Fig. 7f. Obviously, the valid information of these two results is the same. Specifically, the proposed method based on algebraic addition has better ability of suppressing high frequency noise than HCGMP based on algebraic multiplication.

The measuring accuracy of the proposed method can be more accurately demonstrated by measuring a series of planes with known height. During measurement, the planes with height of 6 mm, 15 mm, and 23 mm are measured by $\pi$ phase-shifting FTP, HCGMP and the proposed method respectively, and the results are shown in Table 2.

**Table 2:** Experimental results for different known-height planes (/mm).

$h$	6			15			23		
Method	FTP	HCGMP	Pro	FTP	HCGMP	Pro	FTP	HCGMP	Prop
$\overline{h}$	6.012	5.995	5.996	15.004	14.995	14.997	22.999	22.996	22.997
MAE	0.202	0.195	0.191	0.108	0.082	0.073	0.097	0.077	0.067
RMS	0.222	0.213	0.205	0.132	0.094	0.082	0.119	0.092	0.079

where $h$ indicates the known height of the planes and $\overline{h}$ indicates the average height of the reconstructed planes. Meanwhile, the mean absolute error (MAE) is used to express the accuracy of measurement while the root means square error (RMS) is used to express the repeatability of measurement. It is clearly found that the proposed method has higher measurement precision and repeatable accuracy compared to both $\pi$ phase-shifting FTP and HCGMP.

## Conclusions

A computer-generated moiré profilometry based on algebraic addition is proposed. It introduces another method to generate moiré fringes which inherits the advantages of computer-generated moiré profilometry and figures out a better measuring accuracy. When two fringe patterns are superposed by algebraic multiplication, a small amount of high frequency noise will be introduced into the generated moiré fringes, and some DC component of the fringe pattern and deformed pattern will be remained, which will affect the measuring accuracy to a certain extent. When using positive and the negative AC component of the deformed pattern add to the prestored AC components of the fringe patterns respectively, these problems can be effectively reduced. Experimental results demonstrate that its measuring accuracy is better than both $\pi$ phase-shifting FTP and HCGMP.
